# Pigeon RIG-I Function in Innate Immunity against H9N2 IAV and IBDV

**DOI:** 10.3390/v7072813

**Published:** 2015-07-22

**Authors:** Wenping Xu, Qiang Shao, Yunlong Zang, Qiang Guo, Yongchao Zhang, Zandong Li

**Affiliations:** State key Laboratory for Agrobiotechnology, College of Biological Sciences, China Agricultural University, Beijing 100193, China; E-Mails: xwp120@126.com (W.X.); shaoqiang19880316@126.com (Q.S.); zangyunlong@pagina.com.cn (Y.Z.); fzgq249@163.com (Q.G.); zhangyongchao09@163.com (Y.Z.)

**Keywords:** pigeon, RIG-I, CARDs, IBDV, H9N2

## Abstract

Retinoic acid-inducible gene I (RIG-I), a cytosolic pattern recognition receptor (PRR), can sense various RNA viruses, including the avian influenza virus (AIV) and infectious bursal disease virus (IBDV), and trigger the innate immune response. Previous studies have shown that mammalian RIG-I (human and mice) and waterfowl RIG-I (ducks and geese) are essential for type I interferon (IFN) synthesis during AIV infection. Like ducks, pigeons are also susceptible to infection but are ineffective propagators and disseminators of AIVs, *i.e.*, “dead end” hosts for AIVs and even highly pathogenic avian influenza (HPAI). Consequently, we sought to identify pigeon RIG-I and investigate its roles in the detection of A/Chicken/Shandong/ZB/2007 (H9N2) (ZB07), Gansu/Tianshui (IBDV TS) and Beijing/CJ/1980 (IBDV CJ-801) strains in chicken DF-1 fibroblasts or human 293T cells. Pigeon mRNA encoding the putative pigeon RIG-I analogs was identified. The exogenous expression of enhanced green fluorescence protein (EGFP)-tagged pigeon RIG-I and caspase activation and recruitment domains (CARDs), strongly induced antiviral gene (*IFN-*β, *Mx*, and *PKR*) mRNA synthesis, decreased viral gene (*M* gene and *VP2*) mRNA expression, and reduced the viral titers of ZB07 and IBDV TS/CJ-801 virus strains in chicken DF-1 cells, but not in 293T cells. We also compared the antiviral abilities of RIG-I proteins from waterfowl (duck and goose) and pigeon. Our data indicated that waterfowl RIG-I are more effective in the induction of antiviral genes and the repression of ZB07 and IBDV TS/CJ-801 strain replication than pigeon RIG-I. Furthermore, chicken melanoma differentiation associated gene 5(MDA5)/ mitochondrial antiviral signaling (MAVS) silencing combined with RIG-I transfection suggested that pigeon RIG-I can restore the antiviral response in MDA5-silenced DF-1 cells but not in MAVS-silenced DF-1 cells. In conclusion, these results demonstrated that pigeon RIG-I and CARDs have a strong antiviral ability against AIV H9N2 and IBDV in chicken DF-1 cells but not in human 293T cells.

## 1. Introduction

H9N2 avian influenza virus (AIV) has been the major threat against domestic poultry in China during the last few decades [1]. Despite its weak virulence, H9N2 influenza genotype evolution has facilitated the genesis of the novel H7N9 virus, which has caused the deaths of at least 115 people [2]. Another major problem in the domestic poultry industry is infectious bursal disease (IBD) caused by the infectious bursal disease virus (IBDV) characterized by acute, highly contagious, immunosuppressive disease in the young chicken [3]. Both HPAIV and IBDV cause high morbidity and mortality in chicken poultry but typically do not cause these effects in wild waterfowl and pigeon. As natural reservoirs, ducks and pigeons have been called the “Trojan horse” and “dead end” for susceptibility to infection; however, they are ineffective propagators and disseminators of the virus [4]. This difference in virus resistance among waterfowl, pigeon, and chicken has been ascribed to their individual immune systems, especially their respective innate immune systems.

The recognition of virus-specific pathogen-associated molecular patterns (PAMPs) by host cells is mediated by pathogen recognition receptors (PRRs), including Toll-like receptors (TLRs), Retinoic acid-inducible gene I (RIG-I)-like receptors (RLRs) and Nod-like receptors (NLRs). The RLRs, which consist of RIG-I, MDA5 (melanoma differentiation associated gene 5), and LGP2 (laboratory of genetics and physiology 2), are the major cytoplasmic PRRs of RNA viruses [5]. RIG-I, a 925 amino acid protein, is composed of two *N*-terminal tandem caspase activation and recruitment domains (CARDs), a central DExD/H-box RNA helicase domain, and a Zn^2+^-containing regulatory C-terminal domain (CTD), which is also known as an autorepression domain [6]. During virus infection, prepackaged single-stranded RNA (ssRNA) and double-stranded RNA (dsRNA) generated by viral RNA polymerases, can be recognized by the CTD through the RNA-binding domain [5,7]. Then, RIG-I undergos a conformational change resulting in the exposure of the CARDs to the cytoplasm, which ultimately leads to CARD ubiquitination [6,8]. The activated CARDs interact with the mitochondrial adaptor molecules MAVS, also called interferon-beta promoter stimulator 1(IPS-1), virus-induced signaling adaptor (VISA), and CARD adaptor inducing IFN-β(CARDIF), which leads to TBK1 (TANK-binding kinase 1) and IKK (IkB kinase) activation [9,10,11,12]. TBK1 and IKK activation results in the phosphorylation of the transcription factors IRF-3, IRF-7, and NF-κB [13,14]. The nuclear translocation of these transcription factors, consequently, leads to the transcription of type I interferon (IFN) genes and a set of IFN-induced genes [15].

Ducks’ natural resistance to IAVs and chickens’ susceptibility to IAVs have been linked to a molecular basis of RIG-I, which is absent in the chicken genome [4]. Previous reports have shown that RIG-I derived from waterfowl and mammals induces a strong antiviral response against H9N2 IAV, IBDV and Newcastle disease virus (NDV) [4,16,17,18]. However, relatively little is known regarding the antiviral ability of pigeon RIG-I against these RNA viruses.

In this report, we identified a new analog of pigeon RIG-I. The sequence alignment, subcellular localization, and antiviral response against ZB07 and IBDV TS/CJ-801 strains induced by pigeon RIG-I and CARD overexpression were examined in chicken DF-1 cells and human 293T cells. We also compared the antiviral activities of pigeon RIG-I and CARDs with waterfowl. Finally, the silencing of chicken MDA5/MAVS combined with RIG-I transfection further confirmed that pigeon RIG-I senses AIV H9N2 and IBDV viruses in chicken cells.

## 2. Materials and Methods

### 2.1. Identification and Cloning of Pigeon RIG-I

Two pRIG-I PCR fragments were obtained from cDNA isolated from pigeon (Columba) spleen using the following primers: 253F 5′-GGTTACACAGGACTGGCAGAAGCAAT-3′, 757R 5′-AGGCTGTGCAAGTTCAATCTGGTAG-3′, 1242F 5′-GTTTGACTGCTTCTGTTGGAGTTGGTAAT-3′ and 1892R 5′-CTTAGCAAAGAGAAGAGTGCGAGTCTGT-3′ based on a conserved region of the duck RIG-I sequence. The complete cDNA sequence was obtained via 5′ and 3′ RACE using the 3′/5′-RACE System for Rapid Amplification of cDNA Ends of cDNA Ends (Invitrogen, Waltham, MA, USA). The pRIG-I cDNA sequence has been deposited in the Genebank database (accession no. KP742481). The complete coding region of pigeon RIG-I was amplified using primers in the 5′ UTR 5′-CGGCCGGCAGAGCCCAGCC-3′ and 3′ UTR5′-GTGTAGGAGAGTAATAGATGCACTA-3′ using PrimeStar HS DNA Polymerase (TaKaRa, Otsu, Shiga, Japan). The PCR fragments were then cloned into the pMD™19-T vector (TaKaRa, Otsu, Shiga, Japan) and completely sequenced.

### 2.2. Plasmids

EGFP-tagged duck CARDs/RIG-I and goose CARDs/RIG-I plasmids were reported in a previous study [19]. Homologous pEGFP-N1 and pCMV-N-Flag arms were added to the 5′ and 3′ terminals of pigeon CARDs/RIG-I, human MAVS (hMAVS), and chicken MAVS (cMAVS) by PCR with primers as shown in Table 1. Then, pigeon CARDs/RIG-I, hMAVS, cMAVS were inserted into the mammalian expression vector pEGFP-N1 or pCMV-N-Flag by homologous recombination in accordance with the manufacturer’s instructions (NovoRec^®^ PCR cloning kit, Shanghai, China).

**Table 1:** Primers list for homologous arms.

Name	Direction	Sequence (5′–3′)
ppCARDs-EGFP	Foward	ATTCTGCAGTCGACGGTACATGACCGCGGAGGAGAAGA
	Reverse	CGACCGGTGGATCCCGGGCGAAATGGTGTTCACAAATCAG
ppRIG-I-EGFP	Foward	ATTCTGCAGTCGACGGTACATGACCGCGGAGGAGAAGA
	Reverse	CGACCGGTGGATCCCGGGCGCTGGATGTTTCTTCATCATCA
phMAVS-flag	Foward	CGACGATAAGAGCCCGATGCCGTTTGCTGAAGACAAGACCT
	Reverse	ATTCCTGCAGAAGCTTCTAGTGCAGACGCCGCCGGTAC
pcMAVS-flag	Foward	CGACGATAAGAGCCCGATGGGTTTCGCCGAGGAC
	Reverse	ATTCCTGCAGAAGCTTCTATTTCTGCAATCGTGTGTACACC
ppCARDs-flag	Foward	CGACGATAAGAGCCCGATGACCGCGGAGGAGAAGA
	Reverse	ATTCCTGCAGAAGCTTCTAATGGTGTTCGCAAATCAG

### 2.3. Viruses

ZB07 was kindly provided by Professor Jinhua Liu (College of Veterinary Medicine, China Agricultural University, Beijing, China). Then, the virus was propagated in 10-day-old chicken embryos and the titers were deterimined as 50% tissue culture infectious doses (TCID_50_/mL) by the indirect fluorescent antibody test （IFA） on MDCK cells in the presence of 2 μg/mL tolylsulfonyl phenylalanyl chloromethyl ketone (TPCK)-trypsin (Worthington Biochemical, Lakewood, CA, USA). IBDV TS/CJ-801 strains were amplified in Vero cells, and the titers were determined as TCID_50_ by IFA on DF-1 cells. All of the experiments with ZB07 virus and IBDV TS/CJ-801 strains were conducted under biosafety level 2 (BSL-2) conditions with investigators wearing suitable protective equipment and abiding by general biosafety standards for microbiological and biomedical laboratories of Ministry of Health of the People’s Republic of China (WS 233-2002).

### 2.4. Cell Culture, Infections, and Transfections

DF-1, 293T, Vero and MDCK cells were cultured in Dulbecco’s modified Eagle’s medium (DMEM) basic (1×), supplemented with 0.6 μg/mL penicillin, 60 μg/mL streptomycin and 10% fetal bovine serum (FBS) (Gibco, CA, USA) in 5% CO_2_ at 37 °C. For the infection of the transfected cells, 2.5 × 10^5^ DF-1 and 293T cells were seeded onto 24-well plates and cultured in DMEM basic (1×) plus 10% FBS overnight. Then, the DF-1 and 293T cells were transfected with 0.8 μg of EGFP-tagged plasmids with Lipofectamine 2000 (Invitrogen, Waltham, MA, USA). Then, 24 h later, DF-1 and 293T cells were infected with ZB07 and IBDV TS/CJ-801 strains. TPCK-treated trypsin (0.1 μg/mL) was used in DF-1 cells and in 293T cells (0.5 μg/mL) [20].

### 2.5. Small Interfering RNA Design and Transfection

For knockdown of chicken MDA5 (GenBank number GU570144) and MAVS (GenBank number NM_001012893.1) in DF-1 cells, small interfering RNA (siRNA) and control siRNA were designed and produced by Suzhou GenePharma Co., LTD. All the siRNA sequences are shown in Table 2. For silencing, 2.5 × 10^5^ DF-1 cells were transfected with siRNAs at a 40 nM with 1 μL RNAiMAX regant (Invitrogen, Waltham, MA, USA) according to the manufacturer’s protocol. Then, 24 h after transfection, the cells were infected with ZB07 or IBDV viruses at a multiplicity of infection (MOI) of 1.

**Table 2:** Sequences for small interfering RNA (siRNA).

Name	SiRNA Sequence (5′–3′)
MDA-siRNA1	GCUGCAAGCCAACCAGUAUTT
MDA-siRNA2	GCAUUUACGAAAGGAGUUUTT
MDA-siRNA3	GCAGAACACUUGAAGAAAUTT
MAVS-siRNA1	GCUGUGAGCUCGGAUGUUUTT
MAVS-siRNA2	GCCAAACUCUGCUGCAGAATT
MAVS-siRNA3	GGAUCUGAGCAGGUCUCUUTT
CTR-siRNA	UUCUCCGAACGUGUCACGUTT

### 2.6. Confocal Microscopy

DF-1 and 293T cells were seeded into 12-well chamber slides (CC2 Glass slide, Nunc; Rochester, NY, USA). After 12–16 h, phMAVS-flag and ppRIG-I-EGFP/ppCARDs-EGFP were co-transfected into cells with Lipofectamine 2000 (Invitrogen, Waltham, MA, USA) at a ratio of 1:1. After 24 h, the cells were washed with PBS, fixed with 4% paraformaldehyde, permeabilized with 0.1% triton-x 100 (*v*/*v*) in PBS, and blocked with 0.5% BSA in PBS. The cells were finally stained with anti-flag antibody, Cy3-labeled secondary antibody, and 4′,6-diamidino-2-phenylindole (DAPI), and images were taken on an Olympus confocal microscope.

### 2.7. Western Blotting

Human 293T and chicken DF-1 cells were lysed with Radio immunoprecipitation assay buffer (RIPA buffer) (Thermo Scientific, Waltham, MA, USA). Protein lysates were separated utilizing sodium dodecyl sulfate polyacrylamide gel electrophoresis (SDS-PAGE) and incubated with anti-EGFP (Beijing B&M Biotech Co., Beijing, China) and anti-glyceraldehyde-3-phosphate dehydrogenase (GAPDH) (Beijing CoWin Biotech, Beijing, China) antibodies followed by Horseradish Peroxidase (HRP)-labeled secondary antibodies (Bio-Rad, Hercules, CA, USA) [21].

### 2.8. Quantitative Real-Time PCR

Total RNA was extracted with TRIzol (Invitrogen, Waltham, MA, USA). Then, 1 μg RNA was reverse transcribed into cDNA utilizing the GoScript reverse transcription system (Promega) in a 20 μL reaction system. The cDNA was analyzed utilizing qRT-PCR with SYBR Green Master I (Roche). The primers specific for chicken *GAPDH*, *IFN-*β, *Mx*, and *PKR* and human *GAPDH* and *IFN-*β have been previously described [4,19]. The primers specific for chicken MDA5 and MAVS have been also been previously described [22]. Under the following cycling conditions, qRT-PCR was conducted: 95 °C for 10 min for predenaturation, followed by 40 cycles of 95 °C for 15 s and 60 °C for 1 min. The last step was conducted by 95 °C for 15 s, 60 °C for 15 s and 95 °C for 15 s, which is necessary to acquire a melting curve for the PCR products to confirm the specificity of amplification. The relative mRNA abundances were analyzed utilizing the 2^−ΔΔ*C*t^ method with GAPDH as a reference and plotted as fold changes compared with the mock-treated samples [23].

### 2.9. Antiviral Assay

DF-1 cells transfected with pigeon CARDs/RIG-I and pEGFP-N1 were infected with ZB07, IBDV TS/CJ-801 at an MOI of 0.01. Then, cell supernatants were obtained at different times (24 and 48 h) after infection. The cell cultures were centrifuged at 5000× g for 1 min and the supernatants were stored at −80 °C. The viral titers in the supernatants were determined using TCID_50_ in MDCK and DF-1 cells as previously reported [24].

### 2.10. Cytotoxicity Assays

The cytotoxic effect of transfection on DF-1 cells was measured using the crystal violet staining and MTT assay, respectively. 2.5 × 10^5^ cells were seeded onto 24-well plates overnight, then, DF-1 cells were transfected with pigeon CARDs/RIG-I and pEGFP-N1 respectively. Twenty-four hours and forty-eight hours later, crystal violet staining was used to assess the cell survival. 1 × 10^4^ cells were seeded into 96-well plate overnight. Then, DF-1 cells were transfected with pigeon CARDs/RIG-I and pEGFP-N1 respectively. Twenty-four hours and forty-eight hours later, 50 μL PBS containing MTT (0.5 mg/mL) was added to each well. After incubation at 37 °C for 4 h, the supernatant was removed and 200 μL DMSO was added to each well to solubilize the formazan crystals. Then, absorbance values were measured in a microplate reader (Bio-Rad, Hercules, CA, USA) at 520 nm.

### 2.11. Statistical Analysis

All data analysis was conducted utilizing *SPSS 16.0* software. One-way ANOVA with a post-test was used to test significant differences among different plasmid-transfected groups. *p*-Values ≤ 0.05 were considered significant.

## 3. Results

### 3.1. Cloning and Analysis of Pigeon RIG-I Gene

**Figure 1:**
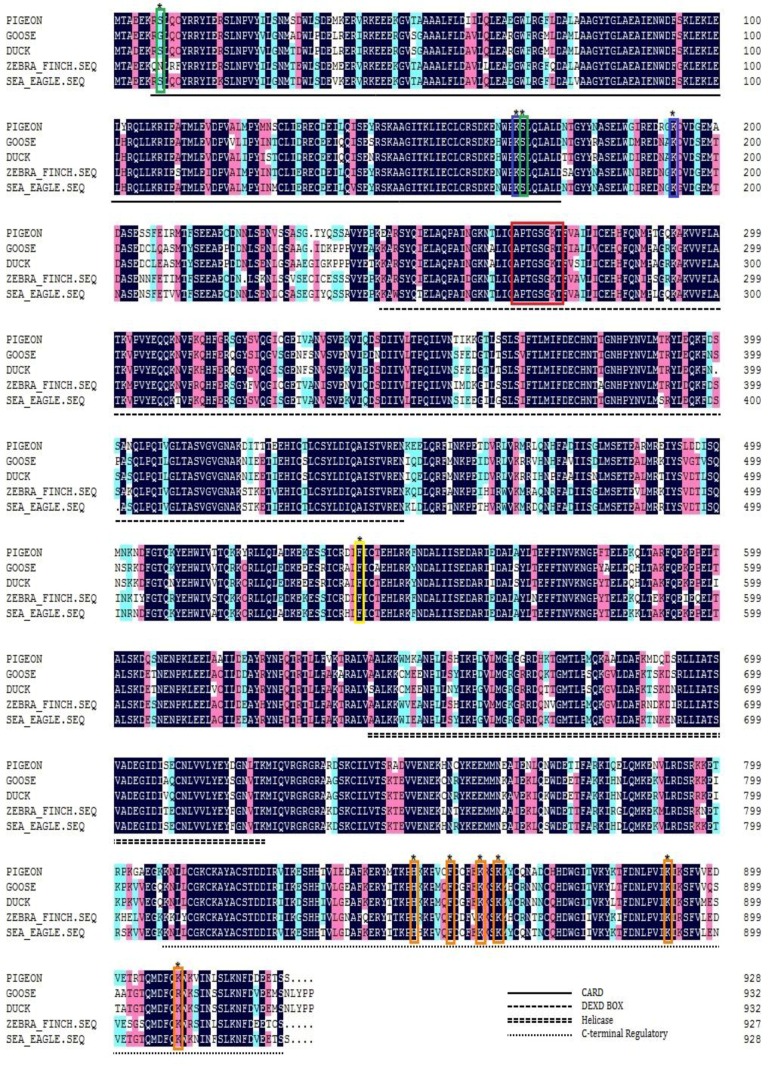
Amino acid alignment of pigeon, goose, duck and zebra finch RIG-I. Black shading indicates amino acid identity, and the green frame indicates the phosphorylation sites. The blue frame denotes ubiquitination sites, and the red frame indicates the ATP-binding motif. The yellow frame indicates the critical residue for the interaction between CARDs and HEL2i, and the orange frame denotes the critical residues for RNA ligand binding.

Given the role of RIG-I in the innate immunity of waterfowl and mammals, we identified a pigeon (*Columba livia*) RIG-I homolog with 78.27% amino acid identity to duck and 82.02% identity to zebra finch RIG-I (Figure 1). Both duck and goose RIG-I contain 933 amino acids, while the pigeon and sea eagle RIG-I contain 928 amino acids. The domain prediction indicated that pigeon RIG-I is composed of two N-terminal CARD domains, a helicase domain, a DEXD/H box helicase domain, and a C-terminal regulatory domain consistent with a mammalian and duck counterpart structure. The hydrophobic core and critical residues of CTD implicated in RNA-ligand binding, H849, F853, K861, K864, K891, and K910, are also completely conserved (Figure 1 orange frame). Residues F540, required for interaction between CARDs and HEL2i to keep the autorepression RIG-I state, is conserved (Figure 1 yellow frame). As a ligand-dependent ATPase, the walker A ATP-binding motif “APPTGSGK” is conserved in pigeon RIG-I (Figure 1 red frame). The residues S8 and S168 phosphorylated by protein kinase C α/β (PKCα/β) and K167 and K193 ubiquitinated by TRIM 25 in duck RIG-I are all present in pigeon RIG-I (Figure 1 green frame and blue frame, respectively).

### 3.2. Overexpression and Cellular Localization of Pigeon RIG-I in Chicken DF-1 Cells and Human 293T Cells

**Figure 2:**
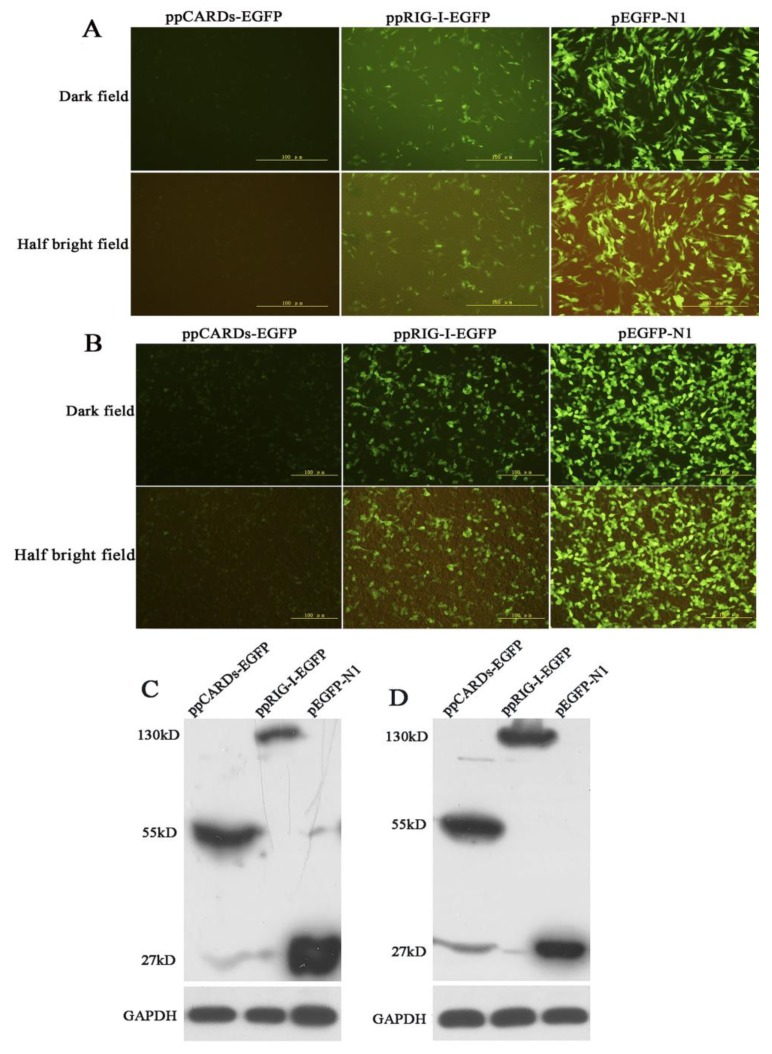
Overexpression of pigeon RIG-I in chicken DF-1 cells and human 293T cells (**A**) DF-1 cells were transfected with pEGFP-N1, ppCARDs-EGFP and ppRIG-I-EGFP. Then, 24 h later, fluorescence microscopy was utilized to examine the EGFP, pCARDs-EGFP and pRIG-I-EGFP expression; (**B**) 293T cells were transfected with pEGFP-N1, ppCARDs-EGFP and ppRIG-I-EGFP. Then, 24 h later, fluorescence microscopy was utilized to examine the EGFP, pCARDs-EGFP and pRIG-I-EGFP expression; (**C**) DF-1 cells were transfected with pEGFP-N1, ppCARDs-EGFP and ppRIG-I-EGFP, and 24 h later cell lysates were separated using SDS-PAGE and probed with anti-EGFP; (**D**) 293T cells were transfected with pEGFP-N1, ppCARDs-EGFP and ppRIG-I-EGFP, and 24 h later cell lysates were separated using SDS-PAGE and probed with anti-EGFP.

DF-1 and 293T cells were transfected with pEGFP-N1, ppCARDs-EGFP and ppRIG-I-EGFP. The fluorescence microscopy analysis indicated that both pigeon CARDs and RIG-I were expressed effectively in DF-1 and 293T cells (Figure 2A,B). Western blotting was performed to detect the expression of the fusion proteins (ppCARDs-EGFP pRIG-I-EGFP and EGFP). As with the fluorescence, Western blot analysis further confirmed pCARDs-EGFP and RIG-I-EGFP expression in chicken DF-1 cells and human 293T cells (Figure 2C,D).

**Figure 3:**
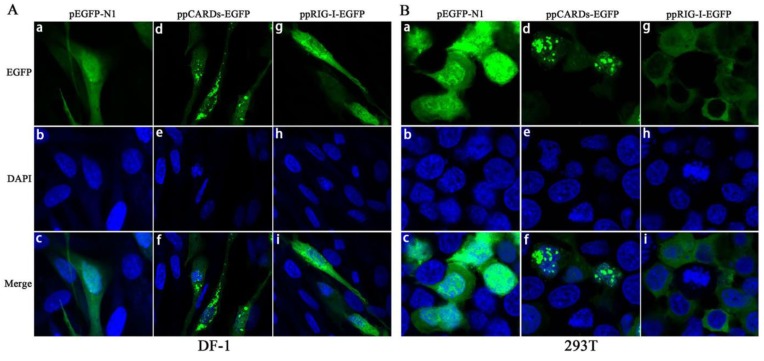
Localization of pigeon CARDs and RIG-I in chicken DF-1 cells and human 293T cells (**A**) DF-1 cells were transfected with pEGFP-N1, ppCARDs-EGFP (ppigeonCARDs-EGFP) and ppRIG-I-EGFP (ppigeonRIG-I-EGFP), and 24 h later, transfected cells were dyed with DAPI, then, the cellular localization of pRIG-I and CARDs was examined via confocal microscopy; (**B**) 293T cells were transfected with pEGFP-N1, ppCARDs-EGFP and ppRIG-I-EGFP, and 24 h later, the transfected cells were dyed with DAPI, then, the cellular localization of pRIG-I and CARDs was examined via confocal microscopy.

Next, we investigated the cellular localization of pigeon CARDs and RIG-I in DF-1 and 293 T cells. For this purpose, we overexpressed EGFP-tagged pCARDs and EGFP-tagged pRIG-I in DF-1 and 293 T cells and examined the localization patterns via confocal microscopy. As shown in Figure 3A,B, the pRIG-I-EGFP dispersoid distributed throughout the cytoplasm of DF-1 and 293T cells, however, pCARDs-EGFP typically localized as dot-like structures in both the cytoplasm and nucleus, implying that pCARDs may directly interact with MAVS and recruit downstream proteins without any virus infection. EGFP-N1 was distributed throughout the cell, including the cytoplasm and the nucleus.

**Figure 4:**
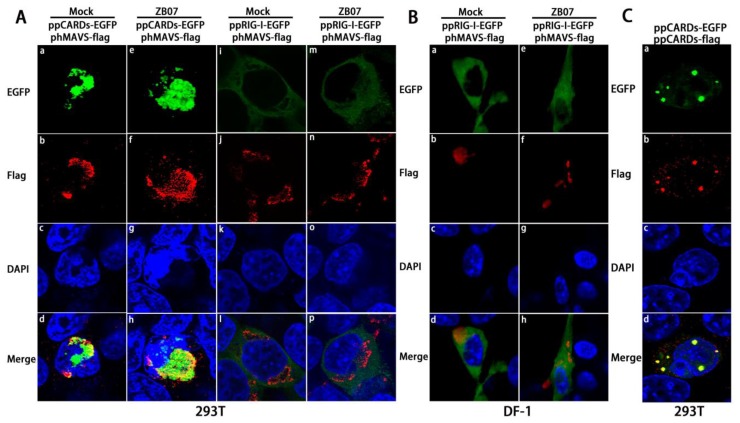
Co-localization of pigeon CARDs with MAVS (**A**) phMAVS-flag and ppRIG-I-EGFP/ppCARDs-EGFP were co-transfected into 293T cells respectively, and 24 h later, transfected cells were infected with ZB07 viruses or mock-treated, then, transfected cells were dyed with anti-flag-antibody and DAPI at 8 h p.i. Then, the cellular localization of pRIG-I, CARDs, and hMAVS was examined via confocal microscopy; (**B**) pcMAVS-flag and ppRIG-I-EGFP were co-transfected into DF-1 cells respectively, and 24 h later, transfected cells were infected with ZB07 viruses or mock-treated, then, transfected cells were dyed with anti-flag antibody and DAPI at 8 h p.i. Then, the cellular localization of pRIG-I and hMAVS was examined via confocal microscopy; (**C**) 293T cells were co-transfected with ppCARDs-EGFP and ppCARDs-flag. 24 h later, transfected cells were dyed with DAPI, anti-flag antibody, and cy3 labled secondary antibody. Then, the cellular localization of pCARDs-EGFP and CARDs-flag was examined via confocal microscopy.

To determine whether pigeon CARDs/RIG-I co-localize with human MAVS, phMAVS-flag and ppCARDs-EGFP/ppRIG-I-EGFP were co-transfected into 293T cells, respectively, followed by ZB07 infection or mock-treated. As shown in Figure 4A(a–h), pCARDs co-localizes with hMAVS with or without virus infection, however, no merged color of pigeon RIG-I and hMAVS was observed (Figure 4A(i–p)). To investigate whether pigeon RIG-I co-localize with chicken MAVS, pcMAVS-flag and ppRIG-I-EGFP were co-transfected into DF-1 cells. pRIG-I-EGFP dispersoid distributed throughout the cytoplasm and partly overlayed with chicken MAVS (Figure 4B). These data suggest that pigeon CARDs can interact with both human and chiken MAVS, while pigeon RIG-I only interact with chicken MAVS. In addition, upon ZB07 infection, full-length pigeon RIG-I didn’t form the dot-like structures as CARDs did (Figure 4B(e–h)), which allows us to speculate that CARDs can gather together to form the dot-like structures. To address this, ppCARDs-EGFP and ppCARDs-flag were co-transfected into 293T cells. Indeed, we observed that pCARDs-EGFP co-localizes with pCARDs-flag (Figure 4C). These results together suggest that pCARDs can gather together as dot-like structure to interact with both human and chicken MAVS, while pRIG-I can’t form the dot-like structure, but it does partly overlay with chicken MAVS, not human MAVS.

### 3.3. Pigeon CARDs and RIG-I Enhanced IBDV- and ZB07- Induced Antiviral Genes Levels in Chicken DF-1 Cells but Not in Human 293T Cells

**Figure 5:**
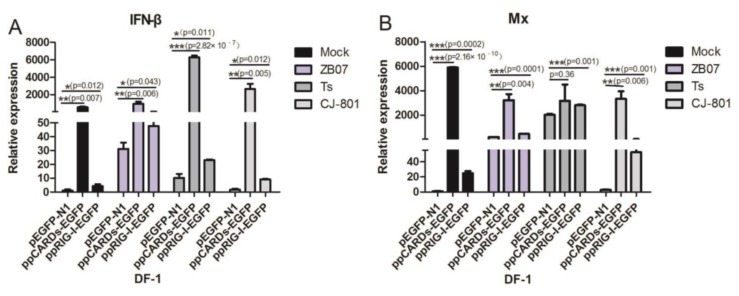

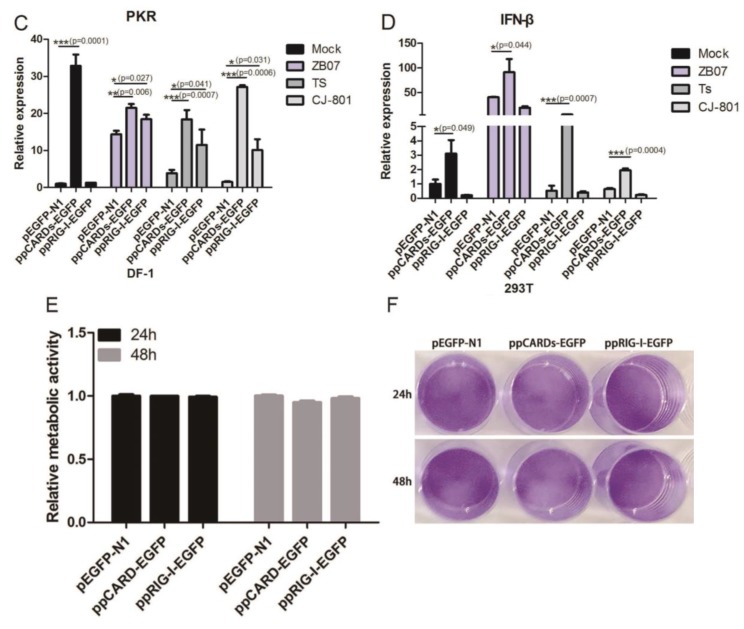
IBDV and ZB07 induced antiviral gene expression in pigeon CARDs and RIG-I transfected DF-1 and 293T cells. (**A–C**) DF-1 cells were transfected with pEGFP-N1, ppCARDs-EGFP and ppRIG-I-EGFP, then, 24 h later, the transfected cells were infected with CJ-801, TS, ZB07 viruses or mock-treated. RNA was extracted and the *IFN-*β, *Mx*, and *PKR* mRNA expression were determined by qRT-PCR at 8 h p.i.; (**D**) 293T cells were transfected with pEGFP-N1, ppCARDs-EGFP and ppRIG-I-EGFP. Then, 24 h later, the transfected cells were infected with CJ-801, TS, ZB07 viruses or mock-treated. RNA was extracted and the *IFN-*β mRNA expression were determined by qRT-PCR at 8 h p.i.; (**E**) DF-1 cells were transfected with ppCARDs-EGFP, ppRIG-I-EGFP, and pEGFP-N1, then, 24 and 48 h later, metabolic activity was measured using the MTT assay; (**F**) DF-1 cells were transfected with ppCARDs-EGFP, ppRIG-I-EGFP, and pEGFP-N1. Then, 24 and 48 h later, crystal violet staining was used to assess cytotoxic effects. Data represent mean ± SEM from three wells per group. Results are representative of three independent experiments. Data represent mean ± SEM from three wells per group. * *p* ≤ 0.05 *vs.* pEGFP-N1; ** *p* ≤ 0.01 *vs.* pEGFP-N1; *** *p* ≤ 0.001 *vs.* pEGFP-N1. Results are representative of two independent experiments.

To determine whether pigeon CARDs and RIG-I induce an antiviral response against IBDV or influenza virus in chicken cells, DF-1 cells were transfected with ppCARDs-EGFP, ppRIG-I-EGFP, or pEGFP-N1, followed by infection with ZB07, IBDV TS/CJ801 or mock-treated. The mRNA levels of *IFN-*β and the IFN-stimulated genes *Mx*/*PKR* were detected by qRT-PCR. The transfection of pigeon CARDs enhanced the *IFN-β* (5-fold), *Mx* (5500-fold), and *PKR* (32-fold) mRNA levels in the mock-treated group (Figure 5A–C), suggesting that pCARDs trigger the type I IFN signaling pathway without virus infection. ZB07, IBDV TS/CJ-801 infection further enhanced pCARDs-induced *IFN-*β expression but decreased the *Mx* and *PKR* mRNA levels (Figure 5A–C), implying that the influenza virus and IBDV interfere with the extremely high expression of *Mx* and *PKR*. The overexpression of pigeon RIG-I caused slightly increased mRNA synthesis of *IFN-*β without affecting *Mx* and *PKR* expression in mock-treated DF-1 cells. However, upon IBDV TS/CJ-801 or ZB07 infection, pRIG-I-EGFP significantly increased *IFN-β*, *Mx,* and *PKR* mRNA expression compared with pEGFP-N1 transfection (Figure 5A–C), suggesting that pRIG-I senses influenza virus and IBDV, and triggers IFN-related genes expression in chicken cells.

To determine whether pigeon CARDs and RIG-I recognize IBDV and ZB07 viruses and induce an antiviral response in human cells, 293T cells were transfected with ppCARDs-EGFP, ppRIG-I-EGFP or pEGFP-N1, followed by IBDV TS/CJ-801 or ZB07 infection. Then, qRT-PCR was conducted to detect *IFN-*β expression. As shown in Figure 4D, pigeon CARDs remarkably increased *IFN-*β mRNA levels with or without infection. However, pRIG-I overexpression blocked *IFN-β* mRNA synthesis in influenza virus-infected 293T cells. In addition, both IBDV TS and IBDV CJ-801 did not increase *IFN-*β expression according to their low infectivity on human cells (Figure 5D).

To investigate whether different IFN responses is due to cytotoxicity of the various plasmids transfection, crystal violet staining and MTT assay were performed to assess the metabolic activity of transfected DF-1 cells. As shown in Figure 5E,F, there was no observable difference in metabolic activity of DF-1 cells transfected with pigeon CARDs/RIG-I and pEGFP-N1, implying that effects of IFN responses is not due to cytotoxicity of the various treatments.

Taken together, these data indicate that pigeon CARDs promote *IFN-*β production with or without influenza virus/IBDV infection in both DF-1 and 293T cells, however, pigeon RIG-I only functions in chicken cells upon influenza virus or IBDV infection.

### 3.4. Pigeon CARDs and RIG-I Transfection Reduced IBDV and Influenza Virus Replication in Chicken DF-1 Cells

**Figure 6:**
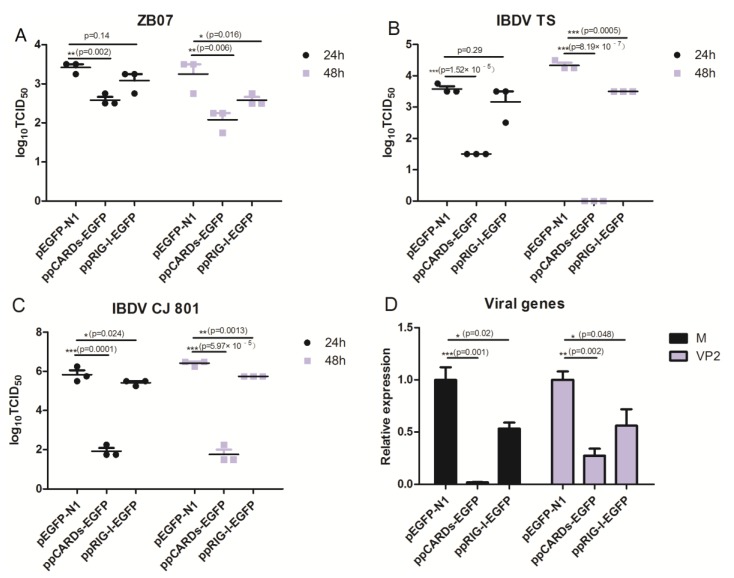
IBDV and ZB07 replication in pigeon CARDs and RIG-I transfected DF-1 cells. (**A**–**C**) DF-1 cells were transfected with pEGFP-N1, ppCARDs-EGFP and ppRIG-I-EGFP, and 24 h later, the transfected cells were infected with CJ-801, TS, and ZB07 viruses (MOI = 0.01). At 24 and 48 h p.i., the viral titers in the cell cultures were determined using TCID50. (**D**) DF-1 cells were transfected with pEGFP-N1, ppCARDs-EGFP and ppRIG-I-EGFP, and 24 h later, the transfected cells were infected with TS and ZB07 viruses. The RNA was extracted from cells for qRT-PCR at 8 h p.i. Data represent mean ± SEM from three wells per group. * *p* ≤ 0.05 *vs.* pEGFP-N1; ** *p* ≤ 0.01 *vs.* pEGFP-N1; *** *p* ≤ 0.001 *vs.* pEGFP-N1. Results are representative of two independent experiments.

Following the observation that pigeon CARDs and RIG-I induced significantly high levels of *IFN-*β or *Mx*, we evaluated the effects of pigeon CARDs and RIG-I on the replication of ZB07 and IBDVTs/CJ-801 viruses. DF-1 cells were transfected with ppRIG-I-EGFP, ppCARDs-EGFP, or pEGFP-N1 and then challenged with ZB07 or IBDVTS/CJ-801 viruses at an MOI of 0.01. The viral titers in the supernatant were determined using TCID_50_ at 24 and 48 h post-infection (p.i.). We observed that pigeon CARDs and RIG-I significantly decreased the ZB07 viral titers of from 3.5 (pEGFP-N1 transfected group) to 2.5 (ppCARDs-EGFP transfected group) or 2.75 (ppRIG-I-EGFP transfected group) log_10_ TCID_50_ at 24 h and 48 h pi (Figure 6A), which is consistent with the *IFN-*β production. The IBDV TS and C-J801 viral titers were decreased to much lower levels than ZB07 in pigeon CARDs and RIG-I-transfected DF-1 cells, especially the ppCARDs-EGFP-transfected group (Figure 6B,C). Furthermore, we analyzed the production of the IBDV *VP2* and ZB07 matrix genes (*M* gene). As with the inhibitory effect of pigeon CARDs and RIG-I on viral titers, *VP2* and *M* gene mRNA expression was significantly lowered in pigeon CARDs and pigeon RIG-I transfected DF-1 cells compared with empty-vector transfection controls (Figure 6D). These observations suggest that pigeon CARDs and RIG-I interfere with viral gene transcription and avian influenza virus and IBDV replication in DF-1 cells.

### 3.5. Waterfowl and Pigeon RIG-I Differ in Their Abilities to Induce Antiviral Response against IBDV and ZB07 in Chicken DF-1 Cells

**Figure 7:**
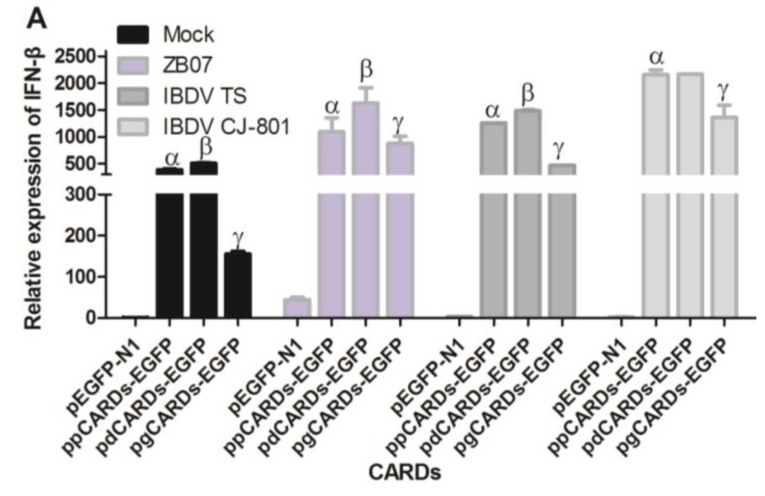

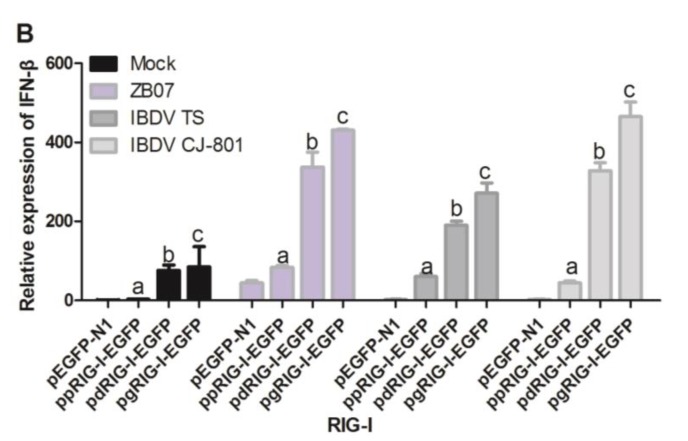
Comparison between waterfowl and pigeon RIG-I in their abilities to induce antiviral response against IBDV and ZB07 in DF-1 cells. (**A**) DF-1 cells were transfected with pEGFP-N1, ppCARDs-EGFP, pdCARDs-EGFP (pduckCARDs-EGFP), pgCARDs-EGFP (pgooseCARDs-EGFP), and 24 h later, the transfected cells were infected with CJ-801, TS, and ZB07 viruses or mock-treated (MOI = 1). qRT-PCR was performed on extracted RNA at 8 h p.i.; (**B**) DF-1 cells were transfected with ppRIG-I-EGFP, pdRIG-I-EGFP (pduckRIG-I-EGFP), pgRIG-I-EGFP (pgooseRIG-I-EGFP), and pEGFP-N1. Then, 24 h later, the transfected cells were infected with CJ-801, TS and ZB07 viruses or mock-treated (MOI = 1). qRT-PCR was performed on extracted RNA at 8 h p.i. Data represent mean ± SEM from three wells per group. ^α^ *p* ≤ 0.05 *vs.* pEGFP-N1, ^β^ *p* ≤ 0.05 *vs.* ppCARDs-EGFP, ^γ^ *p* ≤ 0.05 *vs.* pdCARDs-EGFP, ^a^ *p* ≤ 0.05 *vs.* pEGFP-N1, ^b^ *p* ≤ 0.05 *vs.* ppRIG-I-EGFP, ^c^ *p* ≤ 0.05 *vs.* pdRIG-I-EGFP. Results are representative of two independent experiments.

Our previous report has shown that waterfowl and mammalian RIG-I have different abilities to induce antiviral responses against influenza A virus. To determine the difference in IFN-inducing activity between RIG-I from waterfowl and pigeon, DF-1 cells were transfected with EGFP-tagged plasmids including ppCARDs-EGFP, pdCARDs-EGFP, pgCARDs-EGFP, ppRIG-I-EGFP, pdRIG-I-EGFP, pgRIG-I-EGFP, and pEGFP-N1, which was followed by infection with ZB07, IBDV TS/CJ-801 or mock-treatment. *IFN-*β mRNA expression was detected by qRT-PCR. The results showed that duck CARDs have a stronger ability to induce the expression of *IFN-*β, followed by pigeon CARDs, and goose CARDs with or without infection (Figure 7A). As shown in Figure 7B, goose RIG-I induced the strongest *IFN-*β production with or without virus infection, followed by duck RIG-I, pigeon RIG-I and the empty-vector transfection control, which is consistent with our previous report that duck RIG-I induced less *IFN-*β production than goose RIG-I [19]. These data indicate that although pigeon CARDs induced comparative *IFN-*β levels as duck CARDs, pigeon RIG-I has weaker IFN-inducing activity than both duck RIG-I and goose RIG-I with or without virus infection, which implies that the maintenance of autorepression state or RNA-sensing ability differ in waterfowl and pigeon RIG-I.

### 3.6. Transfection of pRIG-I Restored the Antiviral Response in MDA5-Silencing DF-1 Cells

**Figure 8:**
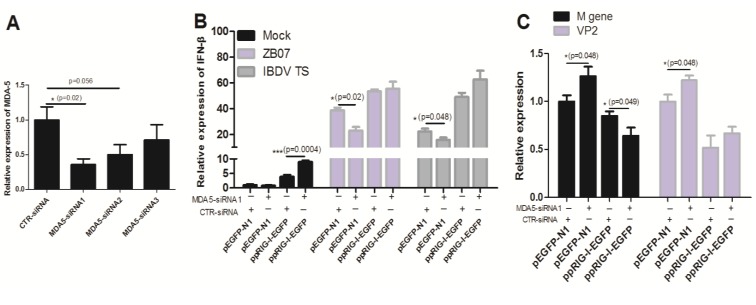
Effect of pRIG-I transfection on IFN synthesis in MDA5-knockdown DF-1 cells. (**A**) siRNA targeting chicken MDA5 and negative control siRNA were transfected into DF-1 cells. Then, 36 h later, the interference efficiency was measured by qRT-PCR; (**B**,**C**) DF-1 cells were co-transfected with pigeon RIG-I/pEGFP-N1 and MDA-siRNA/control siRNA (CTR-siRNA), and 36 h later, the transfected cells were infected with IBDV and ZB07 or mock-treated (MOI = 1). qRT-PCR was performed on extracted RNA at 8 h p.i. Data represent mean ± SEM from three wells per group. * *p* ≤ 0.05 *vs.* CTR-siRNA; ** *p* ≤ 0.01 *vs.* CTR-siRNA; *** *p* ≤ 0.001 *vs.* CTR-siRNA. Results are representative of two independent experiments.

The absence of RIG-I in the chicken genome makes chicken MDA5 a broader spectrum sensor for RNA viruses. Chicken MDA5 is responsible for detecting both influenza virus and IBDV infection in chicken cells. To investigate whether pRIG-I can substitute chicken MDA5 in sensing influenza virus and IBDV, we used RNAi to knockdown MDA5 mRNA expression and transfected DF-1 cells with ppRIG-I-EGFP or pEGFP-N1. As shown in Figure 8A, MDA5-siRNA1/2/3 down-regulated MDA5 mRNA to 35.5%, 49.5%, and 71% of the control group, respectively. MDA5-siRNA1 transfection blocked the ZB07/IBDV TS-induced *IFN-*β mRNA production in pEGFP-N1-transfected DF-1 cells (Figure 8B). However, MDA5-siRNA1 transfection showed no effect on ZB07-induced *IFN-*β mRNA expression in ppRIG-I-EGFP-transfected DF-1 cells (Figure 8B). Furthermore, ppRIG-I-EGFP-transfected DF-1 cells even induced a stronger production of *IFN-*β in MDA5 knockdown cells compared with pEGFP-N1-transfected DF-1 cells during IBDV Ts infection (Figure 8B). The detection of viral *M* gene and *VP2* mRNA expression showed that MDA5-siRNA1 transfection promoted *M* gene and *VP2* mRNA synthesis in pEGFP-N1-transfected DF-1 cells (Figure 8C). However, ppRIG-I-EGFP-transfection reduced *M* gene and *VP2* mRNA to much lower levels compared with pEGFP-N1-transfection with or without MDA5 silencing (Figure 8C). These results imply that pRIG-I can restore the antiviral response in MDA5-silencing DF-1 cells.

### 3.7. MAVS Knockdown Blocked the Antiviral Response Induced by Exogenous pRIG-I in Chicken DF-1 Cells

**Figure 9:**
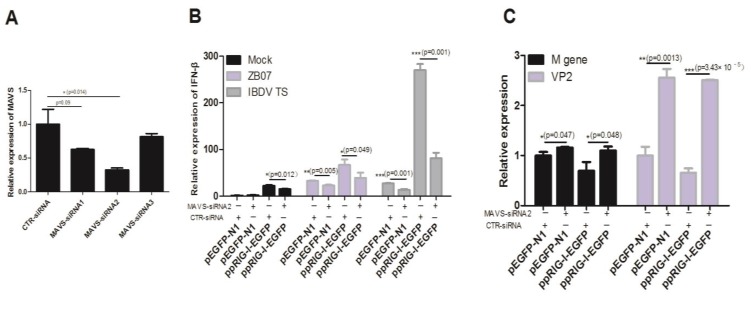
Effect of pRIG-I transfection on antiviral response in MAVS-knockdown DF-1 cells. (**A**) siRNA targeting chicken MAVS and negative control siRNA were transfected into DF-1 cells. Then, 36 h later, the interference efficiency was measured by qRT-PCR; (**B**,**C**) DF-1 cells were co-transfected with ppRIG-I-EGFP/pEGFP-N1 and MAVS-siRNA/control siRNA (CTR-siRNA), 36 h later, the transfected cells were infected with IBDV and ZB07 or mock-treated (MOI = 1). qRT-PCR was performed on extracted RNA at 8 h p.i. Data represent mean ± SEM from three wells per group. * *p* ≤ 0.05 *vs.* CTR-siRNA; ** *p* ≤ 0.01 *vs.* CTR-siRNA; *** *p* ≤ 0.001 *vs.* CTR-siRNA. Results are representative of two independent experiments.

Figure 4 have shown that pCARDs co-localize with MAVS, a major adaptor for both the MDA5 and RIG-I signaling pathway in humans, mice, and chickens. To confirm whether chicken MAVS is responsible for the antiviral effects induced by pRIG-I in DF-1 cells, we used RNAi to knockdown chicken MAVS mRNA expression. As shown in Figure 9A, MAVS-siRNA1/2/3 down-regulated the MDA5 mRNA to 62.5%, 32%, and 81.5% of the control group, respectively. MAVS-siRNA2 transfection significantly reduced ZB07/IBDV-induced *IFN-*β mRNA levels and up-regulated *M* gene and *VP2* mRNA expression in both pEGFP-N1- and ppRIG-I-EGFP- transfected DF-1 cells (Figure 9B,C). ppRIG-I-EGFP transfection increased *IFN-*β mRNA expression and decreased viral gene expression in CTR-siRNA-transfected DF-1 cells. However, no observable differences in *IFN-*β mRNA expression and decreased viral gene expression was detected between pEGFP-N1 and ppRIG-I-EGFP transfection groups combined with MAVS-siRNA (Figure 9B,C). These data suggest that MAVS knockdown blocks the antiviral response induced by chicken MDA5 and exogenous pRIG-I in DF-1 cells.

## 4. Discussion

Here, we establish the presence of RIG-I in pigeon, an ineffective propagator and disseminator of the influenza viruses. Pigeon RIG-I shares 78.27% and 82.02% amino acid identity with its homologs in duck and zebra finch, respectively, suggesting that pigeon RIG-I has a similar role as an RNA virus sensor in innate immunity.

As an effective domain of RIG-I, CARD overexpression alone is sufficient to interact with MAVS, resulting in IFN transcription without infection [4,5,17]. Our data showed that pCARDs co-localized with human MAVS and itself as dot-like structures in 293T cells with or without infection, implying that pCARDs could directly trigger the downstream signaling without virus infection. The IFN response also confirmed this. Pigeon RIG-I dispersoid distributed throughout the cytoplasm of DF-1 cells and could only partly overlay with chicken MAVS, not human MAVS, upon virus infection, indicating that pigeon RIG-I only function in chicken cells but not in human cells.

Transfection of pigeon CARDs into chicken DF-1 cells enhanced the production of IFN-β without infection; however, pRIG-I was not as effective as pCARDs in inducing IFN due to its autorepression by CARDs and the HEL2i interaction when no RNA ligand is present to bind with the *C*-terminal domain. RIG-I recognizes 5′pppRNA and dsRNA (<1 kb) produced during viral RNA replication. Our previous report demonstrated that duck RIG-I increases IFN-β production and represses replication of both influenza A virus (ssRNA virus) and IBDV (dsRNA virus). Chih-chun lee *et al.* [25], reported that chicken MDA5 is involved in the recognition of IBDV. In the current study, pRIG-I significantly increased IFN-β, Mx, and PKR synthesis during IBDV TS/CJ801 or ZB07 infection, implying that pRIG-I is also capable of sensing both influenza virus and IBDV in chicken cells.

Many viruses encode antagonist proteins that function to either directly disrupt the RIG-I signaling pathway or avoid recognition of viral RNA by RIG-I. The RNA binding domain in non-structural (NS1) protein of influenza A viruses can bind with viral RNA to sequester RNA away from RIG-I [26,27]. NS1 can also bind with TRIM25 and RIPLET, preventing RIG-I K63-ubiquitination [28,29]. NS1 can directly block the function of *PKR*. Recently, Swantje *et al.* [30], demonstrated that the pre-packaged PB1/PA complex in influenza virion suppresses early IFN induction by binding with RIG-I. In addition, the PB1-F2 protein of influenza virus can antagonize IFN by interacting with MAVS [31]. Moreover, IBDV employs VP4 protein to block RIG-I signaling via an interaction with glucocorticoid-induced leucine zipper (GILZ) [32]. Consistent with these reports, our current data showed that ZB07, IBDV TS, and IBDV J801 infection down-regulated *Mx* and *PKR* mRNA expression, which may be associated with the antagonist proteins of these viruses.

Our previous study demonstrated that goose RIG-I has no antiviral effects in human cells, and human and mouse RIG-I cannot trigger IFN induction in chicken DF-1 cells upon influenza viruses infection [19]. Consistent with these observations, in this report, we demonstrated that pCARDs promote IFN synthesis in human 293T cells, however, pRIG-I negatively regulates influenza virus-induced IFN expression. Human TRIM25 activates duck CARDs by ubiquitination at residues K167 and K193 in 293T cells [33]. The presence of residues K167 and K193 in pigeon CARDs help to explain the activity of pigeon CARDs in 293T cells. The K858, K861, K888, H847, and F853 residues, which are essential in 5′pppRNA binding by *C*-terminal domain, are also completely conserved in pigeon, indicating that pigeon RIG-I could sense viral RNA. However, the T770, S854/855 residues in the RIG-I *C*-terminal domain, which are essential for phosphorylation regulation by Casein Kinase II in humans, are not present in pigeon and waterfowl RIG-I [34]. The RIPLET, which ubiquitinates Lys849 and Lys851 in the CTD of RIG-I [35,36], is missing in chicken and duck genomes [29,37]. The inactivity of pigeon RIG-I in human cells might ascribe to different regulatory mechanism between birds and mammals. Our previous study demonstrated that duck CARDs/RIG-I and goose CARDs/RIG-I had different ability to induce the production of IFN-β, despite no differences was observed in the inhibition of viruses relication [19]. In this study, we found that pCARDs induced comparative IFN synthesis with duck and goose CARDs, pigeon RIG-I showed weaker IFN-inducing activity compared with duck and goose RIG-I with or without infection, which suggests that pCARDs undergo dephosphorylation, ubiquitination, and interact with MAVS, recruit downstream signaling molecules in chicken cells. However, all three types of RIG-I proteins need to bind with the RNA ligand to undergo a conformational change, which leading to cytoplasmic exposure of CARDs. Therefore, their differences in activities of RNA binding, dephosphorylation and ubiquitination in CTD may result in their different IFN-inducing activities. In addition, a G8S mutant of gCARDs significantly reduced the IFN-inducing activity of gCARDs (Supplementary Figure S1), implying that Gly8 in gRIG-I may set gRIG-I free from the negative regulation of CARD1 phosphorylation; therefore, gRIG-I has a higher IFN-inducing activity than dRIG-I and pRIG-I.

Although influenza virus nucleic acids are mainly primarily recognized by RIG-I, not MDA5 in humans and mice [38], chicken MDA5 plays a critical role in sensing both influenza virus and IBDV in the absence of RIG-I. We used RNAi to prove that pRIG-I restored the antiviral response in MDA5-knockdown DF-1 cells, but not in MAVS-knockdown DF-1 cells, suggesting that pRIG-I is more efficient than chicken MDA5 in sensing influenza virus and IBDV. Furthermore, pigeon RIG-I had a stronger ability to induce *IFN-*β production in MDA5-siRNA transfected DF-1 cells compared with the negative control group during the ZB07/IBDV infection or mock-treatment, which implies that MDA5 competitively binds the downstream protein of the RLR signaling pathway but the pigeon RIG-I has stronger antiviral activity than MDA5. Therefore, the susceptibility to influenza virus or IBDV infection may be due to the absence of RIG-I from chicken genome.

In conclusion, our study indicates that pigeon RIG-I may substitute for chicken MDA5 to induce antiviral response against IBDV and avian influenza virus in chicken cells. Furthermore, pigeon RIG-I plays an essential role in the initiation of type I IFN and IFN-stimulated genes and the reduction of IBDV and ZB07 virus replication. Our results offer evidence that waterfowls and pigeons are more resistant to ZB07 and IBDV infection than chickens.

## Supplementary Files

Supplementary File 1
