# Supplementary material for: Pigeon RIG-I Function in Innate Immunity against H9N2 IAV and IBDV
Source: Viruses. 2015 Jul 22;7(7):4131–51. doi: 10.3390/v7072813 (PMC4517142; doi:10.3390/v7072813)
Supplement: Supplementary File 1 [file viruses-07-02813-s001.pdf]

Supplementary Information

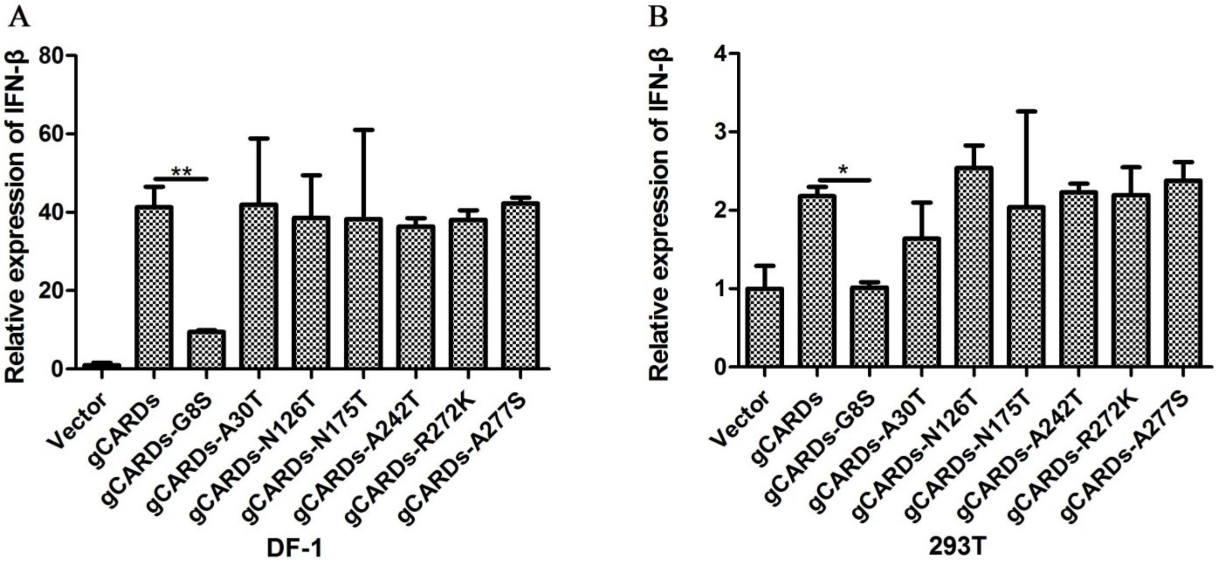

Figure S1. *IFN*-β mRNA levels induced by sites mutagenesis of gCARDs. DF-1 (**A**) and 293T (**B**) cells were transfected with recombinant plasmids for 24 h, then qRT-PCR were performed to detect *IFN*-β mRNA levels. Results are representative of two independent experiments (mean ± SEM). * $p \leq 0.05$ *vs.* gCARDs, ** $p \leq 0.05$ *vs.* gCARDs.
